# Effect of Calcium Sprays on Mechanical Strength and Cell Wall Fractions of Herbaceous Peony (*Paeonia Lactiflora* Pall.) Inflorescence Stems

**DOI:** 10.3390/ijms13044704

**Published:** 2012-04-13

**Authors:** Chengzhong Li, Jun Tao, Daqiu Zhao, Chao You, Jintao Ge

**Affiliations:** 1 College of Horticulture and Plant Protection, Yangzhou University, Yangzhou 225009, China; E-Mails: lichengzhong80@yahoo.com.cn (C.L.); daqiuzhao@126.com (D.Z.); kyzy518529@163.com (C.Y.); gjtwd678@126.com (J.G.); 2 Department of Landscape Architecture and Horticulture Yangzhou Vocational College of Environment and Resources, Yangzhou 225127, China

**Keywords:** calcium, breaking force, cell wall fractions, herbaceous peony, inflorescence stem

## Abstract

Calcium is an essential element and imparts significant structural rigidity to the plant cell walls, which provide the main mechanical support to the entire plant. In order to increase the mechanical strength of the inflorescence stems of herbaceous peony, the stems are treated with calcium chloride. The results shows that preharvest sprays with 4% (*w/v*) calcium chloride three times after bud emergence are the best at strengthening “Da Fugui” peonies’ stems. Calcium sprays increased the concentrations of endogenous calcium, total pectin content as well as cell wall fractions in herbaceous peonies stems, and significantly increased the contents of them in the top segment. Correlation analysis showed that the breaking force of the top segment of peonies’ stems was positively correlated with the ratio of water insoluble pectin to water soluble pectin (*R* = 0.673) as well as lignin contents (*R* = 0.926) after calcium applications.

## 1. Introduction

Herbaceous peonies (*Paeonia lactiflora* Pall.) are popular and well-known ornamental plants [1,2], which originated in China, where most of the cultivars come from and the culture history of which has been known for more than 2000 years [3–5]. Modern peonies cvs have been used for commercial cut flower in some countries for many years, such as USA, New Zealand, Israel, and so on [6,7]. In recent years, peonies’ cut flower production has been expanded in China [8], however, many cultivars of peonies in China have flexible peduncle or inflorescence stem due to weak stem strength and seriously impeded commercial cut flower production.

Mechanical support is provided to the entire plant body mainly by the plant cell walls [5,9], especially the secondary wall, which functions as the skeletal frameworks of plants [10]. The thickness of cell walls in the sclerenchyma and the number of vascular bundles are the important factors that affect the stem mechanical strength [11,12].

Calcium is an essential element as well as a crucial regulator of growth and development in plants [13], and participates in cross-linking negative charges, especially on the carboxylic residues of pectin, imparting significant structural rigidity to the wall [14]. Previous studies indicated that calcium applications could retard the bending of the gerberas inflorescence stems [15,16], and that calcium could improve the stem quality of herbaceous peonies, such as thickness and height [17], but the effect of calcium on the mechanical strength of peonies’ stems is unclear. The cell wall of the herbaceous peony stem is a highly organized composite structure that contains cellulose, hemi-cellulose, lignin, polysaccharides, and proteins [18], but the effect of calcium applications on the cell wall fractions of peonies stems has not yet been described. The aim of this study is to evaluate the effects of calcium on these aspects of herbaceous peonies.

## 2. Results and Discussion

### 2.1. Breaking Force

The effects of calcium applications on the breaking forces in inflorescence stems of herbaceous peonies are shown in Table 1. Quantitative analysis showed that the force required to break the stems which were treated with calcium was significantly higher than that required for the control, it was at least 118.0%, 112.5% and 106.4% in the control stems for the top, middle and bottom segment, respectively. For the top segment, Treatment 3 (4% calcium three times) was the best, and significantly different from other schedules, Treatment 2 (4% calcium twice) was second best, and the effect of Treatment 4 (2% calcium three times) was the worst. For the middle segment and the bottom one, Treatment 3 was both the best, however, other schedules showed no clear difference. So we could reach the conclusion that preharvest sprays with 4% (*w/v*) calcium chloride three times after bud emergence were the best for the mechanical strength of “Da Fugui” peony stems. The effect on the breaking force was significantly reduced when increasing the concentration to 6%, which maybe in excess of the calcium absorption capacity of the plant. In addition, when decreasing the concentration to 2%, insufficient calcium supply may have occurred, regardless of the times of treatment.

The stems were sprayed three times with 4% (*w/v*) calcium chloride. Calcium concentrations together with cell wall materials (CWM) contents in the stems were analyzed to investigate the relationship between the breaking force and these factors.

### 2.2. Calcium Concentrations

Calcium sprays increased calcium concentrations in peony stems regardless of the segment (Table 2). The results were 111.1%, 106.9% and 105.9% in the control stem for the top, middle and bottom segment, respectively. There was significant difference between treatment and the control in the top segment, and none of the calcium sprays led to significant increase of calcium concentration in the middle or the bottom part.

### 2.3. Cell Wall Fractions

#### 2.3.1. Pectin in Different Forms

**Figure 1:**
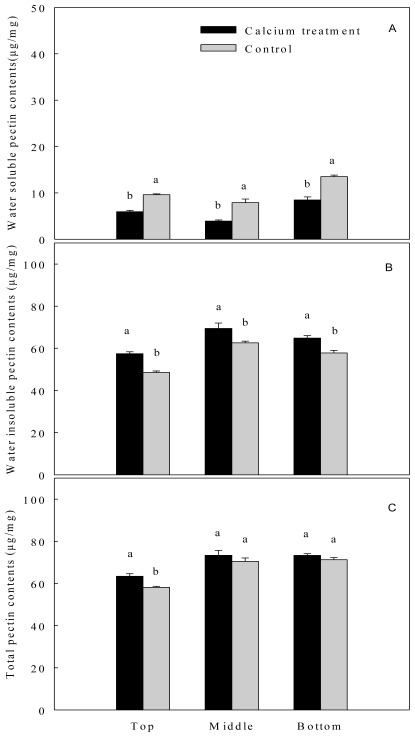
Contents of water soluble pectin (**A**), water insoluble content (**B**) as well as total pectin (**C**) in different segments of herbaceous peony inflorescence stems after sprays with calcium chloride (4%, *w/v*). Different letters above the columns indicate significant difference at *P* = 0.05 between control and calcium treated stems.

For different pectin forms, regardless of the stem segment, calcium sprays reduced the contents of the water soluble pectin significantly (Figure 1A). Meanwhile, it increased the water insoluble pectin contents significantly (Figure 1B). In other words, calcium sprays increased the ratio of water insoluble pectin to water soluble pectin. Correlation analysis showed that the ratio was positively correlated with the breaking force of the top segment peony stems (*R* = 0.673). Calcium applications increased the contents of total pectin of the peony stems (Figure 1C), The contents indicated a great increase in the top segment, but not in the middle or the bottom one.

#### 2.3.2. Cellulose, Hemi-Cellulose and Lignin

**Figure 2:**
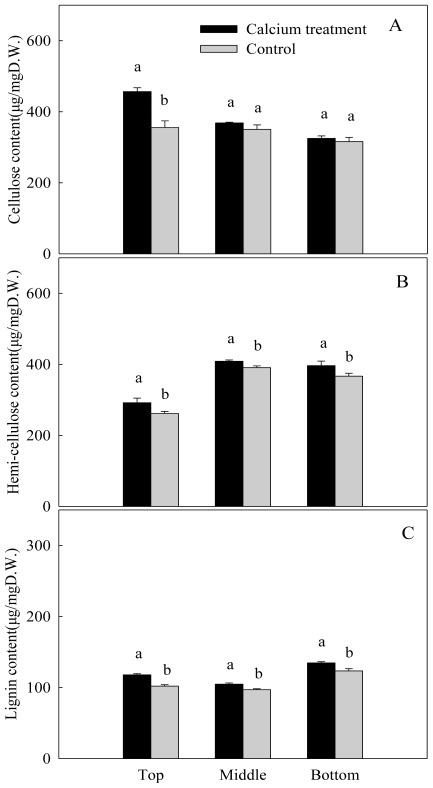
Contents of cellulose (**A**), hemi-cellulose (**B**) and lignin (**C**) in different segment of herbaceous peony inflorescence stems after sprays with calcium chloride (4%, *w/v*). Different letters above the columns indicate significant difference at *P* = 0.05 between control and calcium treated stems.

Calicum sprays significantly increased cellulose contents in the top segment of the peony stems, it was 128.5% in the control stems, but for the middle and the bottom segments, it was 105.2% and 102.9% in the control stems, respectively, and there was no significant difference between treatment and control (Figure 2A). Calcium sprays led to significant increase of hemi-cellulose and lignin contents, regardless of the segment of the stems (Figure 2B,C). Correlation analysis showed that the breaking force of the top segment stems was positively correlated with lignin content (*R* = 0.926) after calcium applications.

## 3. Experimental Section

### 3.1. Plant Material

The experiments were carried out with 3-year-old “Da Fugui”, one of the popular herbaceous peony cultivars in China, which were cultivated in the ornamental plants garden of Yangzhou university, Jiangsu Province, China (32°30′N, 119°25′E). The plants grew well in the natural environment with normal management.

### 3.2. Calcium Treatments

Thirty-six plants of peonies were chosen for treatments. The chosen stems together with the leaves and buds on them were sprayed with calcium chloride (*w/v*) once a week after the bud emergence. The treatments were as follows: Control (water), Treatment 1 (4% calcium once), Treatment 2 (4% calcium twice), Treatment 3 (4% calcium three times), Treatment 4 (2% calcium three times), Treatment 5 (6% calcium three times). All the sprays including the control contained surfactant (0.01% Tween-20). About 1 week after all treatments were complete, we harvested the stems for experiments, the stage of peonies flower opening meanwhile was loose bud, the outer petals soft and loosening, firm inside [8,19].

### 3.3. Breaking Strength Measurement

The inflorescence stems were cut and separated into three segments, 5 cm length from the upper apex or distal of the stem as the top segment, the same length from the proximal and the midst as the bottom and the middle one, respectively. The breaking strength of each segment was measured by the method of Burk *et al*. [20] with a universal digital force testing device (Model NK-2; Huier, Hangzhou, China), and a force was applied manually until the stems were broken. Stems from 6 plants corresponding to each treatment were tested for the breaking force.

### 3.4. Calcium Concentration Determination

Calcium concentration in each part of the inflorescence stems was determined by the method as follows. Each part of the inflorescence stems was cleaned with deionized water and then dried to constant weight at 70 °C. The stems were then ground into powder and sieved. The weighed powder was digested with nitric acid and water (1:1, *v/v*) in the microwave digestion system (MARS 5, CEM, USA). Calcium concentration was determined by atomic absorption spectrophotometer (Solar S4 + Graphite Furnace System 97, Thermo Elemental, USA). The experiment was performed twice.

### 3.5. Cell Wall Materials (CWM) Preparation

The cell wall materials (CWM) were fractioned following the method of Rose *et al*. [21] with some modifications. Briefly, the stems were ground into fine powder in liquid nitrogen and extracted with 95% alcohol, then washed twice with boiling alcohol and methyl alcohol: chloroform (1:1, *v/v*), respectively. Finally, the cell wall residues were dried overnight at 50 °C and used for analysis. The experiment was performed in triplicate.

### 3.6. Uronic Acid, Hemi-Cellulose, Cellulose and Lignin Contents Determination

Pectin content in different forms followed the methods of Manganaris *et al*. [22] for water soluble pectin and Selvendran & O’Neill [23] for water insoluble pectin fraction, and the uronic acid was determined by a colorimetric assay [24], using galacturonic acid as the standard. Total pectin content was the sum of pectin content in both fractions. Hemi-cellulose content was determined by the phenol colorimetric assay [25] and the cellulose content was measured by the anthrone colorimetric assay [26]. Lignin content was determined following the method of Müse *et al*. [27]. Measurements were done in triplicate.

### 3.7. Statistical Analysis

Statistical analyses were conducted using SPSS 16.0. Differences in breaking strength and the content of cell wall fractions among treatments were analyzed by LSD test at a significance level of 0.05, one-way ANOVA.

## 4. Conclusions

Our data indicated that preharvest calcium sprays provided an increase in calcium contents in herbacous peonies stems which corresponded to the increase of breaking force to afford better mechanical support to the plant body. The results were in line with the research by Hui *et al*. [28] except for the concentrations and spraying times of calcium chloride, and partly inconsistent with the research by Yu *et al*. [17]. The mainly reason was the peony cultivars were different.

Calcium is one of the most important components to confer strength to cell walls [29], and over 60% of calcium compounds in plants were associated with pectin [30,31]. Calcium is accumulated in the middle lamella [32], bound inside and between pectin and other components forming a structure named “egg box” [33,34]. Calcium makes cell walls rigid; if calcium ions were at a high level, the pectin chains would be cross-linked and aggregated, and the wall maximally rigidified [14].

Our data indicated that the ratio of water insoluble pectin to water soluble pectin raised after calcium application, which had high positive correlation with the breaking force of the peonies stems. Studies on the effectiveness of spraying calcium in peach storage also found that calcium application could fortify the rigidity of cell walls of the peach by high water insoluble pectin [35,36]. Our data indicated that calcium sprays were more effective in the top segment of herbaceous peonies stems regarding breaking force, which had a high positive correlation with lignin contents. Previous studies on Arabidopsis [20,37], crops [5,10,11,38] and ornamental plants [39] indicated that the mechanical strength of the stems was highly correlated with the content of the secondary cell wall components, such as cellulose, hemicelluloses, and lignin, which partly agreed with our studies on herbaceous peony stems.

## Figures and Tables

**Table 1:** Breaking force measurement in herbaceous peony inflorescence stems with different calcium spray schedules.

Spray schedules	Breaking Force (*N*)		

	Top	Middle	Bottom
Control (water)	21.7 ± 0.8 e	53.6 ± 3.1 c	106.7 ± 4.5 c
Treatment 1 (4% calcium once)	28.4 ± 1.4 bc	60.3 ± 1.9 b	113.9 ± 3.0 b
Treatment 2 (4% calcium twice)	29.6 ± 1.1 b	62.2 ± 1..5 b	115.3 ± 3.9 b
Treatment 3 (4% calcium three times)	32.9 ± 1.1 a	67.3 ± 2.6 a	125.5 ± 4.9 a
Treatment 4 (2% calcium three times)	25.6 ± 1.6 d	63.0 ± 1.8 b	113.6 ± 2.8 b
Treatment 5 (6% calcium three times)	27.0 ± 2.1 cd	61.6 ± 2.8 b	113.5 ± 2.8 b

Data are mean values ± SE for 6 stems. Different letters behind the figures in each column indicate significant difference at *P* = 0.05 among different calcium spray schedules.

**Table 2:** Calcium concentrations (unit: μg/g D.W.) in herbaceous peony inflorescence stems after sprays with calcium chloride (4%, *w/v*).

Treatments	Calcium concentration		
	
	Top	Middle	Bottom
Control	14.40 ± 0.21 b	13.10 ± 0.27 a	11.81 ± 0.81 a
Calcium sprays	16.03 ± 1.25 a	14.04 ± 1.23 a	12.50 ± 0.15 a

Data are mean values ± SE for 2 stems. Different letters with the columns indicate significant difference at *P* = 0.05 between control and calcium treated stems.
